# Intra-abdominal pressure and abdominal perfusion pressure in cirrhotic patients with septic shock

**DOI:** 10.1186/2110-5820-2-S1-S4

**Published:** 2012-07-05

**Authors:** Hasan M Al-Dorzi, Hani M Tamim, Asgar H Rishu, Abdulrahman Aljumah, Yaseen M Arabi

**Affiliations:** 1 Department of Intensive Care Medicine, King Saud bin Abdulaziz University for Health Sciences, King Abdulaziz Medical City, Riyadh, 11426, Saudi Arabia; 2 Department of Epidemiology and Biostatistics, King Saud bin Abdulaziz University for Health Sciences, King Abdulaziz Medical City, Riyadh, 11426, Saudi Arabia; 3 Department of Hepatobiliary Sciences and Liver Transplantation, King Saud bin Abdulaziz University for Health Sciences, King Abdulaziz Medical City, Riyadh, 11426, Saudi Arabia

**Keywords:** liver cirrhosis, sepsis, compartment syndrome, septic shock, ascites, mortality.

## Abstract

**Background:**

The importance of intra-abdominal pressure (IAP) and abdominal perfusion pressure (APP) in cirrhotic patients with septic shock is not well studied. We evaluated the relationship between IAP and APP and outcomes of cirrhotic septic patients, and assessed the ability of these measures compared to other common resuscitative endpoints to differentiate survivors from nonsurvivors.

**Methods:**

This study was a *post hoc *analysis of a randomized double-blind placebo-controlled trial in which mean arterial pressure (MAP), central venous oxygen saturation (ScvO_2_) and IAP were measured every 6 h in 61 cirrhotic septic patients admitted to the intensive care unit. APP was calculated as MAP - IAP. Intra-abdominal hypertension (IAH) was defined as mean IAP ≥ 12 mmHg, and abdominal hypoperfusion as mean APP < 60 mmHg. Measured outcomes included ICU and hospital mortality, need for renal replacement therapy (RRT) and ventilator- and vasopressor-free days.

**Results:**

IAH prevalence on the first ICU day was 82%, and incidence in the first 7 days was 97%. Compared to patients with normal IAP, IAH patients had significantly higher ICU mortality (74.0% vs. 27.3%, *p *= 0.005), required more RRT (78.0% vs. 45.5%, *p *= 0.06) and had lower ventilator- and vasopressor-free days. On a multivariate logistic regression analysis, IAH was an independent predictor of both ICU mortality (odds ratio (OR), 12.20; 95% confidence interval (CI), 1.92 to 77.31, *p *= 0.008) and need for RRT (OR, 6.78; 95% CI, 1.29 to 35.70, *p *= 0.02). Using receiver operating characteristic curves, IAP (area under the curve (AUC) = 0.74, *p *= 0.004), APP (AUC = 0.71, *p *= 0.01), Acute Physiology and Chronic Health Evaluation II score (AUC = 0.71, *p *= 0.02), but not MAP, differentiated survivors from nonsurvivors.

**Conclusions:**

IAH is highly prevalent in cirrhotic patients with septic shock and is associated with increased ICU morbidity and mortality.

## Background

Cirrhotic patients with septic shock (SS) represent a unique group with different presentation, pathophysiology and prognosis compared to other critically ill patients [1-6]. Because of the presence of ascites [7], which is often complicated by spontaneous bacterial peritonitis [8], increased intra-abdominal pressure (IAP) occurs frequently in these patients. Studies have demonstrated that intra-abdominal hypertension (IAH) is common in critically ill patients [9,10] and is associated with multiple organ dysfunction [11-14] and increased mortality [15]. Additionally, liver dysfunction is a significant IAH risk factor in these patients [16]. In cirrhotic patients, Luca et al. found that mechanically increasing IAP in 14 patients with portal hypertension led to deleterious effects such as increased azygos blood flow and decreased cardiac output and hepatic blood flow [17]. However, little is known about the clinical significance of IAH in critically ill cirrhotic patients. Moreover, it is not clear whether abdominal perfusion pressure (APP) is a good resuscitation endpoint in cirrhotics.

Therefore, we studied the occurrence of IAH in cirrhotic patients admitted with SS both on admission and during the intensive care unit stay and assessed its association with mortality. The ability of IAP and APP compared to other commonly used resuscitation endpoints to differentiate survivors from nonsurvivors was also assessed.

## Methods

### Patients and setting

This study was conducted in a 21-bed closed medical-surgical ICU of a tertiary care hospital with an active liver transplant service. The ICU was staffed 24/7 by board-certified intensivists. In 2007, there were 1,118 ICU admissions with a mean Acute Physiology and Chronic Health Evaluation (APACHE) II score [18] of 24.4 and an APACHE II-adjusted standardized mortality ratio of 0.97. This study was a *post hoc *analysis of a randomized double-blinded placebo-controlled trial that studied the effect of low-dose hydrocortisone on the outcomes of cirrhotic patients admitted to the ICU with SS. The trial, performed between April 2004 and October 2007, included 75 adult patients and was approved by the hospital's Institutional Review Board [19]. It excluded patients who had hypovolemic or hemorrhagic shock, known adrenal insufficiency, prior steroid use or contraindication for steroids [19]. It showed that hydrocortisone improved hemodynamics, but not mortality, and was associated with increased side effects [19]. The current study included the 61 patients who had repeated IAP measurements.

### IAP measurement

The standard method for indirectly measuring IAP is to measure the intra-vesicular pressure [20]. For the clinical trial, IAP was measured by trained critical care nurses using the modified Kron technique [21] every 6 h for up to 7 days after ICU admission. Briefly, the bladder drainage system was clamped just distal to the connection of the urinary catheter to the drainage bag. An 18-gauge needle was then inserted into the sampling port and connected via a sterile tube to the pressure transducer using two three-way stopcocks. A standard infusion bag of normal saline was attached to one stopcock, and a 60-ml syringe was connected to the second stopcock. Sterile saline (50 to 100 ml) was injected into the bladder. Measurements were taken at end-expiration while patients were in complete supine position and with the transducer zeroed at the symphysis pubis level.

### Patient management

Patients were managed using a goal-directed therapy for the treatment of septic shock [22]. This entailed achieving mean arterial pressure (MAP) ≥ 65 mmHg, central venous pressure (CVP) ≥ 8 mmHg and central venous oxygen saturation (ScvO_2_) ≥ 70% using fluids, vasopressors, inotropes and/or blood transfusion. No goals were set for IAP and APP such that the management of IAH was left to the discretion of the attending intensivist.

### IAH definitions

The World Society of the Abdominal Compartment Syndrome (http://www.wsacs.org), in its latest consensus statement [23], defined IAH as sustained or repeated IAP of ≥ 12 mmHg. Likewise, abdominal compartment syndrome (ACS) was defined as IAP > 20 mmHg in combination with at least one new end-organ failure, which can be identified by a Sequential Organ Failure Assessment (SOFA) sub-score ≥ 3 [23]. In this study, we calculated the mean of the first four IAP measurements done on each of the first 7 days of ICU stay. Patients with IAP ≥ 12 mmHg were considered to have IAH. Those with IAP ≥ 20 mmHg were considered to have ACS. In addition, APP was calculated by subtracting IAP from simultaneous MAP measurements. Mean APP < 60 mmHg was considered abnormal [24].

### Collected data

We collected the following demographic and clinical information: age, gender, body mass index, liver cirrhosis etiology, Child-Pugh score, admission APACHE II score, SOFA [25], presence of ascites on physical exam, diagnosis of spontaneous bacterial peritonitis and hepatic encephalopathy on ICU admission, admission hemoglobin, albumin, serum lactate, creatinine, bilirubin and ammonia, international normalized ratio (INR), the ratio of the partial pressure of arterial oxygen to the fraction of inspired oxygen (PaO_2_/FiO_2_) and requirement for mechanical ventilation. We also extracted data on MAP, CVP, ScvO_2_, IAP and APP.

### Outcome measures

The primary outcome measure was ICU mortality. The secondary outcomes were hospital mortality, ventilator-free days, vasopressor-free days, duration of mechanical ventilation, ICU and hospital length of stay and the need for renal replacement therapy (RRT), including continuous veno-venous hemofiltration or intermittent hemodialysis. RRT was initiated at the discretion of the attending intensivist in consultation with the nephrology department.

### Statistical analysis

Data were analyzed using SAS software (version 8.0; SAS Institute, Cary, NC, USA). Continuous data were presented as mean with standard deviation (SD), whereas categorical ones were summarized as absolute and relative frequencies (percent). Demographic and physiologic variables were compared among the different IAP groups using the Student's *t *test or chi square/ Fisher's exact test based on whether the variable was continuous or categorical. Stepwise multiple logistic regression was used to study IAH predictors with the following independent variables: APACHE II score, presence of ascites and of encephalopathy, mechanical ventilation, number of transfused PRBC units on day 1, net fluid balance on day 1, INR and bilirubin. To examine the association between IAP and APP and different endpoints, we used multivariate logistic regression analysis to adjust for the following variables: age, APACHE II, Child-Pugh score, requirement for mechanical ventilation, creatinine, INR, fluid balance on day 1 and hydrocortisone therapy. Moreover, the ability of IAP and APP to discriminate survivors from nonsurvivors was compared to other standard hemodynamic endpoints (MAP and ScvO_2_) and APACHE II using the receiver operating characteristic (ROC) curves. Youden index was calculated to assess the best IAP and APP cut-offs that discriminate survivors from non survivors [26]. Additionally, survival analysis was performed using Kaplan-Meier curves to examine the time-dependent mortality difference stratified by different hemodynamic targets (MAP ≥ 65 and < 65 mmHg, ScvO_2 _≥ 70% and < 70% and IAP and APP ≥ and < the best cut-offs on the ROC curve analysis).

## Results

### Prevalence and incidence of IAH and characteristics of the patients according to IAH

The studied patients had the following characteristics (Table 1): age = 59.0 ± 13.0 years, 58% were men, hepatitis C was the most common cause of cirrhosis (44%) with mean Child-Pugh score of 11.6 and most patients had ascites (94%) and hepatic encephalopathy (76%). All patients were on vasopressors, and most (90%) required mechanical ventilation. On the first ICU admission day, mean IAP was 16.7 ± 3.9 mmHg, with 50 patients (82%) having IAH, and 9 patients (15%) having ACS. In addition, 70% of patients had mean APP < 60 mmHg. During the first 7 days of ICU stay, 97% of patients developed IAH, and 39% had ACS.

**Table 1:** Characteristics of patients according to mean IAP and APP on the first ICU admission day.

	All patients	IAP^a ^< 12 mmHg	IAP^a ^≥ 12 mmHg	*p *value	APP^a ^≥ 60 mmHg	APP^a ^< 60 mmHg	*p *value
	*N *= 61	*N *= 11	*N *= 50		*N *= 18	*N *= 43	
Age in years, mean ± SD	59.8 ± 12.2	63.6 ± 7.4	59.0 ± 13.0	0.26	59.0 ± 14.2	60.2 ± 11.5	0.74
Male sex, *N *(%)	36 (59.0)	7 (63.7)	29 (58.0)	1.00	7 (38.9)	18 (41.9)	0.83
Body mass index (Kg/m^2^), mean ± SD	26.8 ± 5.9	26.5 ± 6.3	26.9 ± 5.9	0.87	26.3 ± 6.5	27.0 ± 5.8	0.70
Etiology of liver cirrhosis, *N *(%)							
Hepatitis C	30 (49.2)	8 (73.0)	22 (44.0)		7 (39.0)	23 (53.0)	
Hepatitis B	17 (27.9)	2 (18.0)	15 (30.0)	0.27	6 (33.0)	11 (26.0)	0.56
Others	14 (23.0)	1 (9.0)	13 (26.0)		5 (28.0)	9 (21.0)	
Child-Pugh score, mean ± SD	11.6 ± 1.6	11.2 ± 1.4	11.6 ± 1.6	0.39	11.2 ± 1.9	11.7 ± 1.4	0.29
APACHE II score, mean ± SD	30.1 ± 7.5	23.9 ± 7.7	31.5 ± 6.7	0.002	29.9 ± 7.8	30.2 ± 7.4	0.91
SOFA score, mean ± SD	14.9 ± 3.7	12.5 ± 4.5	15.4 ± 3.3	0.02	13.9 ± 3.7	15.3 ± 3.7	0.1
SOFA renal sub-score	2.2 ± 1.4	2.0 ± 1.7	2.3 ± 1.3	0.52	1.7 ± 1.4	2.5 ± 1.3	0.06
SBP, *N *(%)	19 (31.1)	7 (63.6)	12 (24.0)	0.03	5 (27.8)	14 (32.6)	0.71
Encephalopathy, *N *(%)	42 (68.9)	4 (36.4)	38 (76.0)	0.03	11 (61.1)	31 (72.1)	0.28
Ascites, *N *(%)	58 (95.1)	11 (100)	47 (94.0)	0.05	16 (88.9)	42 (97.7)	0.26
Mechanically ventilated, *N *(%)	51 (83.6)	6 (54.5)	45 (90.0)	0.01	16 (88.9)	35 (81.4)	0.71
PaO_2_/FiO_2 _ratio (mmHg), mean ± SD	252 ± 141	261 ± 129	250 ± 144	0.83	194 ± 117	277 ± 144	0.04
Norepinephrine dose at inclusion (μg/kg/min), mean ± SD	0.35 ± 0.35	0.20 ± 0.29	0.39 ± 0.35	0.11	0.26 ± 0.34	0.39 ± 0.35	0.21
Creatinine^b ^(μmol/l), mean ± SD	289 ± 165	299 ± 176	286 ± 165	0.81	239 ± 147	309 ± 170	0.13
INR, mean ± SD	2.8 ± 1.4	2.6 ± 1.5	2.8 ± 1.4	0.71	2.4 ± 0.7	2.9 ± 1.6	0.08
Bilirubin^b ^(μmol/l), mean ± SD	340.3 ± 284.3	146.0 ± 193.5	383.1 ± 284.6	0.01	313.3 ± 305.2	351.7 ± 278.1	0.63
Ammonia^b ^(μmol/l), mean ± SD	110.2 ± 101.1	74.7 ± 41.1	118.0 ± 108.8	0.03	111.6 ± 167.2	109.6 ± 57.4	0.96
Albumin (g/l), mean ± SD	32.5 ± 7.1	33.8 ± 8.1	32.2 ± 6.9	0.50	31.5 ± 5.4	32.9 ± 7.7	0.48
Hemoglobin (g/dl), mean ± SD	8.6 ± 1.6	9.4 ± 1.6	8.4 ± 1.6	0.27	8.3 ± 1.5	8.7 ± 1.6	0.42
Lactate level^b ^(mmol/l), mean ± SD	4.4 ± 4.3	3.7 ± 4.6	4.5 ± 4.2	0.59	2.9 ± 2.2	5.0 ± 4.8	0.02
Fluid intake in day 1 (ml), mean ± SD	4,356 ± 2,574	3,993 ± 1,877	4,436 ± 2,712	0.61	4,015 ± 2,486	4,499 ± 3,336	0.51
Fluid balance in day 1 (ml), mean ± SD	3,177 ± 2,919	1,544 ± 2,980	3,537 ± 4,335	0.04	2,368 ± 2,913	3,516 ± 2,887	0.16
MAP day 1 (mmHg), mean ± SD	70.7 ± 7.2	58.8 ± 5.8	59.4 ± 8.1	0.83	63.3 ± 7.1	57.6 ± 7.3	0.007
CVP day 1 (mmHg), mean ± SD	15.5 ± 6.2	12.3 ± 4.0	16.2 ± 6.4	0.054	14.1 ± 6.5	16.1 ± 6.1	0.28
IAP day 1 (mmHg), mean ± SD	15.3 ± 4.7	9.0 ± 2.2	16.7 ± 3.9	< 0.0001	12.8 ± 3.8	16.3 ± 4.7	0.008
APP day 1 (mmHg), mean ± SD	55.5 ± 7.7	59.4 ± 6.2	54.6 ± 7.8	0.06	64.7 ± 5.4	51.6 ± 4.7	< 0.0001
Randomization, *N *(%)							
Hydrocortisone	32 (52.5)	4 (36.4)	28 (56.0)	0.24	14 (77.8)	18 (41.9)	0.01
Placebo	29 (47.5)	7 (63.6)	22 (44.0)		4 (22.2)	25 (58.1)	

IAP ≥ 12 mmHg indicates intra-abdominal hypertension, and APP < 60 mmHg indicates abdominal hypoperfusion. IAP, intra-abdominal pressure; APP, abdominal perfusion pressure; SD, standard deviation; APACHE, Acute Physiology and Chronic Health Evaluation; SOFA, Sequential Organ Failure Assessment; SBP, spontaneous bacterial peritonitis; MAP, mean arterial pressure; CVP, central venous pressure; INR, international normalized ratio; PaO_2_/FiO_2_, the ratio of the partial pressure of arterial oxygen (PaO_2_) to the fraction of inspired oxygen (FiO_2_). ^a^Mean values on the first ICU day. ^b^To convert creatinine to mg/dl, divide by 88.4; bilirubin to mg/dl, divide by 17.1; ammonia to μg/dl, divide by 0.587; lactic acid to mg/dl, divide by 0.111.

The characteristics of the study population according to IAP (< 12 vs. ≥ 12 mmHg) and APP (< 60 vs. ≥ 60 mmHg) are shown in Table 1. On the stepwise logistic regression analysis, none of the studied variables was associated with IAH, including fluid balance on day 1 (OR, 1.28/l increment; 95% CI, 0.90 to 1.81).

### Hemodynamic endpoints

**Figure 1:**
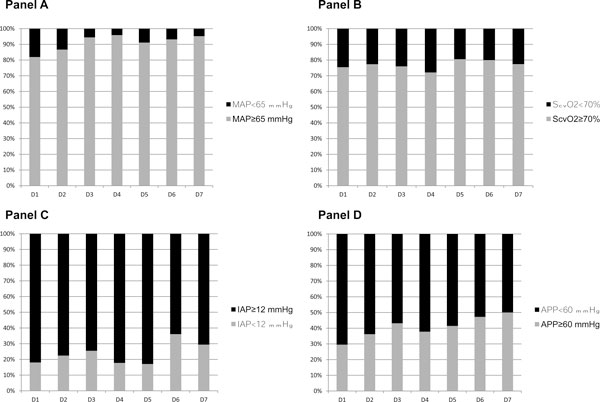
**Day-by-day percentages of cirrhotic patients with septic shock**. With mean arterial pressure ≥ 65 mmHg vs. < 65 mmHg **(A)**, central venous oxygen saturation ≥ 70% vs. < 70% **(B)**, intra-abdominal pressure ≥ 12 mmHg vs. < 12 mmHg **(C) **and abdominal perfusion pressure ≥ 60 mmHg vs. < 60 mmHg **(D) **in the first 7 days of intensive care unit stay.

Figure 1 describes four hemodynamic indices (MAP, ScvO_2_, IAP and APP) observed in the first 7 days of ICU admission. Whereas MAP ≥ 65 mmHg and ScvO_2 _≥ 70% were achieved in most patients (e.g., 86% and 77% of patients, respectively, on day 2), IAP < 12 mmHg and APP ≥ 60 mmHg were present in fewer patients (23% and 36%, respectively, on day 2). ICU survivors and nonsurvivors had similar day 1 ScvO_2 _(75 ± 8% and 78 ± 11%, respectively, *p *= 0.35) and MAP (71 ± 7 mmHg vs. 70 ± 7 mmHg, respectively, *p *= 0.42). On the other hand, ICU survivors had significantly lower day 1 IAP (13 ± 4 mmHg vs. 16 ± 5 mmHg, *p *= 0.015) and higher APP (59 ± 7 mmHg vs. 54 ± 8 mmHg, *p *= 0.02) compared to nonsurvivors.

### Relationship between IAH and outcomes

**Figure 2:**
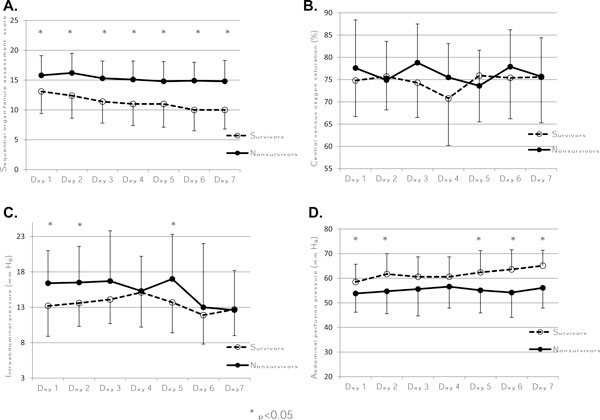
**Evolution of the different variables studied**. Sequential organ failure assessment score **(A)**, central venous oxygen saturation **(B)**, intra-abdominal pressure **(C) **and abdominal perfusion pressure **(D) **during the first 7 days of intensive care unit stay in ICU survivors and nonsurvivors. Error bars represent standard deviations.

The ICU and hospital mortality for all patients were 65.6% and 88.5%, respectively. Table 2 describes the outcomes of patients according to the presence or absence of IAH on the first ICU day. Although the ICU length of stay was similar in both groups, IAH patients had significantly fewer vasopressor- and ventilator-free days than the non-IAH group and tended to require RRT more often (78.0% vs. 45.5%, *p *= 0.06). ICU mortality was almost three times higher in the IAH group (74.0% vs. 27.3%, *p *= 0.005). However, the hospital mortality was not statistically different between the two groups (92.0% vs. 72.7%, *p *= 0.10). The association between APP and outcomes followed a similar pattern but was not statistically different. Figure 2 describes the evolution of SOFA, ScvO_2_, IAP and APP in ICU survivors and nonsurvivors during ICU stay, and shows clear separation for the SOFA score, APP and, to a lesser extent, IAP, but not ScvO_2_.

**Table 2:** Outcomes of cirrhotic patients with septic shock according to the occurrence of intra-abdominal hypertension and abdominal hypoperfusion.

	IAP^a ^< 12 mmHg	IAP^a ^1 ≥ 12 mmHg	*p *value	APP^a ^≥ 60 mmHg	APP^a ^< 60 mmHg	*p *value
	*N *= 11	*N *= 50		*N *= 18	*N *= 43	
ICU mortality, *N *(%)	3 (27.3)	37 (74.0)	0.005	9 (50)	31 (72.1)	0.10
Hospital mortality, *N *(%)	8 (72.7)	46 (92.0)	0.10	15 (83.3)	39 (90.7)	0.41
Duration of MV in days, mean ± SD	4.8 ± 7.5	8.2 ± 6.7	0.14	9.6 ± 7.1	8.9 ± 6.4	0.70
Ventilator-free days, mean ± SD	15.3 ± 11.4	4.7 ± 7.2	0.01	7.4 ± 8.7	6.3 ± 9.2	0.65
Vasopressor-free days, mean ± SD	10.5 ± 8.4	4.6 ± 7.1	0.02	8.4 ± 8.6	4.5 ± 7.1	0.07
ICU LOS (days), mean ± SD	11.1 ± 8.0	9.8 ± 6.0	0.56	11.0 ± 6.7	9.6 ± 6.2	0.45
Hospital LOS (days), mean ± SD	31.1 ± 16.8	21.1 ± 14.6	0.05	23.6 ± 10.3	22.7 ± 17.1	0.81
Requirement for RRT, *N *(%)	5 (45.5)	39 (78)	0.06	11 (61.1)	33 (76.7)	0.21
RRT-free days, mean ± SD	10.8 ± 12.7	5.0 ± 7.5	0.17	7.4 ± 8.9	5.5 ± 8.8	0.44

IAP ≥ 12 mmHg indicates intra-abdominal hypertension, and APP < 60 mmHg indicates abdominal hypoperfusion. IAP, intra-abdominal pressure; APP, abdominal perfusion pressure; RRT, renal replacement therapy; MV, mechanical ventilation; LOS, length of stay. ^a^Mean values on the first ICU day.

For patients with IAP < 12 mmHg, ICU mortality was 50.0% for the hydrocortisone-treated group compared to 14.3% for the placebo group, *p *= 0.49. In IAH patients, ICU mortality was 64.3% for the hydrocortisone-treated patients compared to 86.4% for the placebo group, *p *= 0.08. Randomization to either hydrocortisone or placebo did not affect ICU mortality for patients with APP < or ≥ 60 mmHg.

### IAP and APP as predictors of outcomes

Stepwise multivariate logistic regression analysis (Table 3) showed that IAH was significantly associated with increased ICU mortality (OR, 12.20; 95% CI, 1.92 to 77.31, *p *= 0.008) and need for RRT (OR, 6.78; 95% CI, 1.29 to 35.70, *p *= 0.02), but not hospital mortality (OR, 6.83; 95% CI, 0.86 to 54.12, *p *= 0.07). The relationship between abdominal hypoperfusion (APP < 60 mmHg) and various outcomes did not reach statistical significance (Table 3).

**Table 3:** Stepwise multivariate logistic regression analysis for three outcomes: mortality in the ICU, hospital and need for RRT.

Variable	Intra-abdominal hypertension		Variable	Abdominal hypoperfusion	
	Odds ratio	95% Confidence interval		Odds ratio	95% Confidence interval
ICU mortality					
Intra-abdominal hypertension	12.20	1.92 to 77.31	Abdominal hypoperfusion	2.86	0.74 to 11.11
Age	1.02	0.97 to 1.08	Age	0.98	0.92 to 1.04
INR	3.60	1.29 to 10.06	INR	2.67	1.02 to 7.00
			APACHE II score	1.14	1.02 to 1.26
Hospital mortality					
Intra-abdominal hypertension	6.83	0.86 to 54.12	Abdominal hypoperfusion	2.44	0.38 to 6.67
Age	1.07	0.99 to 1.17	Age	1.05	0.97 to 1.14
INR	6.2	1.07 to 38.5	INR	6.36	0.92 to 44.22
			APACHE II score	1.11	0.98 to 1.26
Renal replacement therapy					
Intra-abdominal hypertension	6.78	1.29 to 35.70	Abdominal hypoperfusion	2.78	0.68 to 11.11
Age	0.92	0.92 to 1.04	Age	0.96	0.90 to 1.02
INR	2.06	0.98 to 4.32	APACHE	1.14	1.04 to 1.25
Child-Pugh score	0.51	0.30 to 0.90	Child-Pugh score	0.63	0.40 to 0.98
Creatinine	1.00	1.00 to 1.01			

Variables used in the model were age, Child-Pugh score, Acute Physiology and Chronic Health Evaluation II score, creatinine, INR, fluid balance on day 1, hydrocortisone therapy and either the presence of intra-abdominal hypertension (mean intra-abdominal pressure ≥ 12 vs. < 12 mmHg) or abdominal hypoperfusion (mean abdominal perfusion pressure < 60 vs. ≥ 60 mmHg) on first ICU admission day. Variables with *p *< 0.1 on stepwise multivariate logistic regression analysis are reported in the table. APACHE, Acute Physiology and Chronic Health Evaluation; INR, international normalized ratio; RRT, renal replacement therapy.

### Resuscitation indices as predictors of ICU mortality

**Figure 3:**
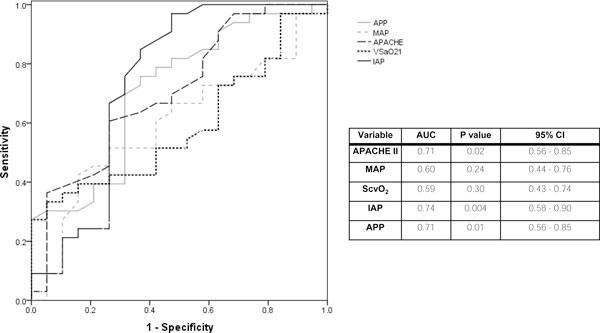
**Receiver operating characteristic curve analysis for predictors of intensive care unit mortality**. The variables studied are Acute Physiology and Chronic Health Evaluation (APACHE) II score, mean arterial pressure, central venous oxygen saturation, intra-abdominal pressure and abdominal perfusion pressure. These variables, except for APACHE II score, were the mean of measurements taken every 6 h on the first admission day to the intensive care unit.

Figure 3 shows the ROC curves of four resuscitation indices (MAP, ScvO_2_, IAP and APP) and APACHE II. Of the four indices, only IAP and APP had significant discrimination (areas under the curve 0.74 [95% CI, 0.58 to 0.90] and 0.71 [95% CI, 0.56 to 0.85], respectively) whereas MAP and ScvO_2 _did not. IAP = 12.4 mmHg and APP = 54.7 mmHg were found to be the cut-offs with the highest calculated Youden index (0.38 and 0.36, respectively) and, hence, the best to discriminate survivors from nonsurvivors.

**Figure 4:**
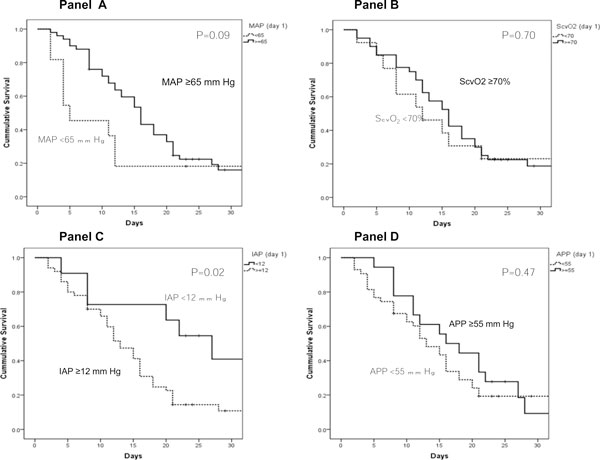
**Kaplan-Meier survival curves**. For cirrhotic septic patients with mean arterial pressure ≥ 65 and < 65 mmHg **(A)**, central venous oxygen saturation ≥ 70% and < 70% **(B)**, intra-abdominal pressure ≥ 12 and < 12 mmHg **(C) **and abdominal perfusion pressure ≥ 55 and < 55 mmHg **(D)**. These resuscitation endpoints were the mean of measurements taken every 6 h on the first admission day to the intensive care unit.

Figure 4 describes the Kaplan-Meier curves of patients having the following resuscitation indices on the first ICU day (MAP ≥ 65 vs. < 65 mmHg, ScvO_2 _≥ 70% vs. < 70%, IAP ≥ 12 vs. < 12 mmHg and APP ≥ 55 vs. < 55 mmHg) and shows the presence of a significant survival difference based on IAP < 12 vs. ≥ 12 mmHg.

## Discussion

The main findings of this study were the following: IAH was common in cirrhotic patients presenting with SS; IAH was an independent predictor for the need for RRT and of ICU mortality, and finally, IAP discriminated better between survivors and nonsurvivors than MAP and ScvO_2_.

Literature on IAH in patients with liver cirrhosis is scarce. One study showed that mechanically increasing IAP by 10 mmHg in 14 patients with portal hypertension led to a reduction in hepatic blood flow by 20% (*p *< 0.05) [17]. Another study found that increased IAP leads to an increase in variceal pressure, radius, volume and wall tension, which may trigger variceal bleeding [27]. These findings suggest that increased IAP contributes to acute decompensation of liver function and to various cirrhosis-related complications, which support our findings.

In this study, IAH was defined as mean IAP ≥ 12 mmHg at which physiologic and clinical derangements usually occur [9,23]. APP < 60 mmHg was considered as an indicator of abdominal hypoperfusion as this pressure has been shown to correlate well with survival from IAH and ACS [28]. This followed the recommendation of the International Conference of Experts on Intra-abdominal Hypertension and Abdominal Compartment Syndrome [24]. We found that 82% of cirrhotic patients with SS had IAH, and most patients (70%) had reduced APP (< 60 mmHg) on the first ICU admission day. This is higher than the IAH prevalence in general ICU patients and could be explained by the high prevalence of ascites in cirrhotics. A multicenter, prospective one-day point-prevalence study conducted in 13 ICUs of six countries demonstrated that IAH was present in > 50% of all surgical and medical critically ill patients hospitalized for > 24 h [9]. Interestingly, we found that most patients with spontaneous bacterial peritonitis (64%) had normal IAP. This may be explained by the fact that these patients might have undergone therapeutic and diagnostic abdominal paracentesis before ICU admission.

Whether IAH is a marker of illness severity or a cause of critical illness or clinical deterioration remains unclear. Malbrain et al. found that the development of IAH during ICU stay, and not IAH at ICU admission, was a risk factor for mortality in a general population of critically ill patients [16]. In our study, IAH on admission was associated with a higher APACHE II score but was also a strong independent predictor of the need for RRT and ICU mortality on multivariate logistic regression analysis. The potential pathophysiologic changes responsible for these effects are multiple. Various organs, inside and outside the abdomen, can be adversely affected by increased IAP [12]. For example, IAH is associated with increased intracranial pressure [29], cardiac dysfunction [30], respiratory failure [31], splanchnic hypoperfusion [13] and acute renal insufficiency [32]. These are probably the results of mechanical and nonmechanical effects of increased IAP [33]. In association with massive fluid resuscitation, the acute intestinal permeability syndrome, which is part of global capillary leak, may lead to multi-organ failure [33]. In addition, IAH is associated with a proinflammatory state [28]. In a study of ten rats, IAH of 20 mmHg caused a significant increase in tumor necrosis factor α and interleukin-6 after 30 min and an increase in interleukin-1b after 60 min [28]. This state may serve as a second insult for the development of multiple organ failure. Of note, IAH was significantly associated with ICU, but not hospital mortality. Explanations include that ICU survivors had advanced cirrhosis and died later during hospitalization because of disease progression with or without Do-Not-Resuscitate orders, or did not receive liver transplantation because of organ shortage or being unfit. Additionally, because our study included the first ICU admission only, the ICU mortality represents the first ICU admission mortality, while hospital mortality includes mortality during all ICU admissions and ward admissions.

Because of the adverse effects of IAH on various organs, lowering IAP and using APP as a resuscitation endpoint appear to be appealing management strategies. A retrospective study of 144 surgical patients treated for IAH (IAP ≥ 15 mmHg) with resuscitation and, if needed, with open abdominal decompression surgery found that APP was able to discriminate survivors from nonsurvivors better than MAP and lactate [34]. In our study, the ROC curves showed that IAP and APP, but not MAP or ScvO_2_, predicted survival, raising the question whether IAP and APP are better resuscitative endpoints than the traditional ones. The ROC curve analysis also suggested that IAP < 12 mmHg and APP > 55 mmHg should be considered therapeutic targets in cirrhotics with SS. Additionally, we found that, in most patients in whom traditional resuscitative endpoints were achieved (MAP ≥ 65 mmHg, ScvO_2 _≥ 70%), IAP and APP were not. Achieving the target MAP and ScvO_2_ alone might not be enough in this population.

IAH management is recommended in critically ill patients [24] even though strong evidence of its benefit remains lacking. A prospective observational study of 478 consecutive surgical patients requiring an open abdomen for the management of IAH or ACS showed a significant decrease in hospital mortality after the implementation of a comprehensive management algorithm for IAH and ACS [35]. In 23 cirrhotic patients with hepatorenal syndrome, infusion of 200 ml of 20% human albumin solution followed by large-volume abdominal paracentesis resulted in IAP reduction from a median of 22 to 9 mmHg and a significant increase in creatinine clearance during the subsequent 12 h from 23 ml/min to 33 ml/min (*p *= 0.002) [36]. Moreover, creatinine clearance remained elevated for up to several days afterwards [36]. Medical interventions that have been suggested to treat IAH include sedation, neuromuscular blockade, gastric or colonic decompression, hypertonic fluids or colloids, forced diuresis and hemofiltration with ultrafiltration [24]. Supporting organ function with vasopressors and judicious goal-directed fluid resuscitation to maintain an APP ≥ 50 to 60 mmHg has also been advocated [24,35]. More invasive procedures including percutaneous catheter decompression or drainage and surgical decompression might be helpful, especially when IAP exceeds 25 mmHg [35]. Our study did not assess the physiologic effects of interventions such as abdominal paracentesis or the prognostic implications of IAH management.

Our findings should be interpreted in the light of the strengths and limitations of the study. Strengths include the prospective data collection, the IAP measurements every 6 h by trained critical care nurses and the clearly defined clinical outcomes. However, there are some limitations. First, it was a single-center retrospective study. Second, it had a small sample size. Nevertheless, our patient population, cirrhotic patients with SS, is interesting and merits additional research. Third, it lacked data on the effect of ascites evacuation. Fourth, the intra-vesicular pressure was measured using 50 to 100 ml of sterile saline, which is too large and has been shown to overestimate intra-vesicular pressure [37,38]. The current recommendation is to use a maximum of 25 ml [23]; however, the study began before such a recommendation was published. Fifth, the use of the symphysis pubis as the zero reference point is outdated. It is currently recommended to zero the transducer at the mid-axillary line level [23,39]. Although the study was conducted over a relatively long period (2004 to 2007), clinical management was standardized and followed a strict study protocol and, therefore, unlikely to have affected outcomes.

In conclusion, IAH was at least a marker of increased ICU morbidity and mortality in cirrhotic patients with SS. Based on the physiologic effects of increased IAP, IAH might even be a contributing factor to the development of organ dysfunction and death in them. Whether the IAH management improves the outcome of this patient population needs to be further studied in a randomized controlled trial. This future research is especially important considering that the prognosis of cirrhotic patients who develop critical illness remains poor despite aggressive therapy and advances in intensive care.

## List of abbreviations

ACS: abdominal compartment syndrome; APACHE: Acute Physiology and Chronic Health Evaluation; APP: abdominal perfusion pressure; CI: confidence interval; CVP: central venous pressure; IAP: intra-abdominal pressure; IAH: intra-abdominal hypertension; INR: international normalized ratio; MAP: mean arterial pressure; OR: odds ratio; ROC: receiver operating characteristic; RRT: renal replacement therapy; ScvO_2_: central venous oxygen saturation; SD: standard deviation; SOFA: Sequential Organ Failure Assessment; SS: septic shock; WSACS: World Society on Abdominal Compartment Syndrome.

## Competing interests

The authors declare that they have no competing interests.

## Authors' contributions

HMA conceived and designed the study, analyzed and interpreted the data and drafted the manuscript. HMT and AHR acquired and analyzed the data and made revisions in the manuscript. AA interpreted the data and made revisions in the manuscript. YMA, the principal investigator of the original trial, conceived and designed the study, interpreted data and drafted the manuscript. All authors made critical revisions, have read and approved the final manuscript.

## Authors' information

HMA, MD is an Intensive Care Medicine consultant at King Abdulaziz Medical City and an assistant professor of the College of Medicine, King Saud bin Abdulaziz University for Health Sciences. HMT, MPH, PhD is an associate professor of Epidemiology and Biostatistics at the College of Medicine, King Saud bin Abdulaziz University for Health Sciences. AHR, MBBS is a research coordinator of the Intensive Care Department at King Abdulaziz Medical City. AA, MD is the division head and consultant of the Hepatobiliary Sciences and Liver Transplantation division of King Abdulaziz Medical City and an assistant professor of the College of Medicine, King Saud bin Abdulaziz University for Health Sciences. YMA, MD, FCCP, FCCM is the Intensive Care Department chairman of King Abdulaziz Medical City and an associate professor of the College of Medicine, King Saud bin Abdulaziz University for Health Sciences.
